# A Computer Program for Calculation of Approximate Embryo/Fetus Radiation Dose in Nuclear Medicine Applications

**DOI:** 10.4274/Mirt.99

**Published:** 2012-04-01

**Authors:** Tuncay Bayram, Bircan Sönmez

**Affiliations:** 1 Karadeniz Technical University Science Faculty, Department of Physics, Trabzon, Turkey; 2 Sinop University Science and Art Faculty, Department of Physics, Sinop, Turkey; 3 Karadeniz Technical University, School of Medicine, Department of Nuclear Medicine, Trabzon, Turkey

**Keywords:** nuclear medicine, radiation dosage, Pregnancy

## Abstract

**Objective:** In this study, we aimed to make a computer program that calculates approximate radiation dose received by embryo/fetus in nuclear medicine applications.

**Material and Methods:** Radiation dose values per MBq-1 received by embryo/fetus in nuclear medicine applications were gathered from literature for various stages of pregnancy. These values were embedded in the computer code, which was written in Fortran 90 program language.

**Results:** The computer program called nmfdose covers almost all radiopharmaceuticals used in nuclear medicine applications. Approximate radiation dose received by embryo/fetus can be calculated easily at a few steps using this computer program.

**Conclusion:** Although there are some constraints on using the program for some special cases, nmfdose is useful and it provides practical solution for calculation of approximate dose to embryo/fetus in nuclear medicine applications.

**Conflict of interest:**None declared.

## INTRODUCTION

Many women are exposed to ionizing radiation each year during their pregnancy for both diagnostic and therapeutic purposes. Ionizing radiation is known to cause harm on the embryo and fetus. Potential adverse outcomes related to radiation exposure during pregnancy include teratogenicity, genetic damage, intrauterine death and increased risk of malignancy development. The risk of each effect depends on the gestational age at the time of exposure and the absorbed radiation dose level. During the preimplantation (0-10 days after conception) and implantation (10-14 days) stages, radiation may cause death of the zygote or embryo. This appears to be an “all or none” effect and a surviving embryo will go on to develop normally. Organogenesis starts 3-5 weeks after conception. Radiation risks are most significant during organogenesis and in the early fetal period somewhat less in the second trimester and least in the third trimester (1). 

In nuclear medicine, patients are administered varying quantities of different radiopharmaceuticals for the diagnosis or treatment of disease. These radiopharmaceuticals will expose patients to ionizing radiation. Although during pregnancy it is strongly recommended to avoid diagnostic or therapeutic nuclear medicine procedures, in cases of clinical necessity or when the physician does not know about the pregnancy, patients may undergo certain nuclear medicine procedures. For these procedures, the absorbed dose to embryo/fetus depends upon deposit of radiopharmaceuticals in the maternal tissues, placental transfer of radiopharmaceuticals, retention and distribution of radiopharmaceuticals, the physical half-life, type of decay products and photon irradiation from radionuclides in the maternal tissues and placenta (2,3). 

In nuclear medicine applications, the doses received by embryo/fetus for therapeutic purposes are much higher than those of diagnostic purposes. Most nuclear medicine procedures do not cause large fetal doses because Tc-99m labeled radiopharmaceuticals are frequently used during pregnancy and they lead to a fetal absorbed dose of less than 10 mSv (4), while some radiopharmaceuticals labeled with I-131 can pose significant fetal risks (2,5). Using hospital records, Doll et al. concluded that radiation doses even in the order of 10 mSv received by the fetus in utero produce a consequent increase in the risk of getting childhood cancer (6). In addition, a report by the International Commission on Radiological Protection (ICRP) advises that diagnostic and therapeutic procedures causing exposure of the abdomen in women likely to be pregnant should be avoided unless there are strong clinical indications (7). 

Although there is some evidence that radiation doses in the order of 10 mSv can cause an increase of getting childhood cancer risk as mentioned above, general aspects about fetal dose can be summarized: If fetal dose is less than 10 mSv, there is no evidence supporting the increased incidence of any deleterious developmental effects on the embryo/fetus at diagnostic doses; if fetal dose is between 10 mSv and 100 mSv, the additional risk of gross congenital malformations, mental retardation, intrauterine growth retardation and childhood cancer is thought to be low compared to the baseline risk; if fetal dose exceeds 100 mSv, the lower limits for threshold doses for effects such as mental retardation, diminished IQ and school performance fall. 

When radiopharmaceuticals administered to pregnant patients either out of clinical necessity or by accident, an accurate estimate of radiation absorbed dose to the fetus is necessary. International Commission on Radiological Protection (ICRP) has the responsibility for calculating dose from intakes of radionuclides by both embryo and fetus as well as members of the public and workers. ICRP published a series of publications giving dose coefficients for public (8,9,10) as well as embryo and fetus (5,11) based on some standardized kinetic models and, in some cases, including knowledge of the amount of radiopharmaceutical crossover, as measured in animal or human studies. Also, reviews on data available on the embryo/fetus dose caused by radiopharmaceuticals used in nuclear medicine applications can be found in literature (5,11,12,13). 

There is a computer program available, called nmdoses (14) which calculates radiation dose to organs and gonads for some commonly used diagnostic and therapeutic radiopharmaceuticals in nuclear medicine. However, it does not include fetus dose calculation for nuclear medicine applications. In addition, the calculation of doses received by embryo/fetus is grueling and time consuming for physicians and health physicists. So, the aim of this study is to make a computer program which calculates approximate embryo/fetus radiation dose at a few steps.

## MATERIALS AND METHODS

In this computer program, for each radiopharmaceutical, radiation dose values of embryo or fetus given as mGy for per MBq to various stages (12,13) were used. These values were embedded in the computer code, which was written in Fortran 90 program language. After compiling of the code nmfdose program was ran on Windows XP, Vista and 7 Operating Systems and checked. This program is called nmfdose.

## RESULTS

Some radiopharmaceuticals included by nmfdose program are listed in Table 1. User can calculate approximate embryo or fetus doses at four steps using nmfdose program. Because it is distributed in zip format, firstly the user should extract nmfdose program to any directory. In order to run the program, user should follow the instructions listed below:

1. Select a radionuclide

2. Select a radiopharmaceutical

3. Select a gestational age

4. Input the activity of selected radionuclide in MBq 

**Figure 1:**
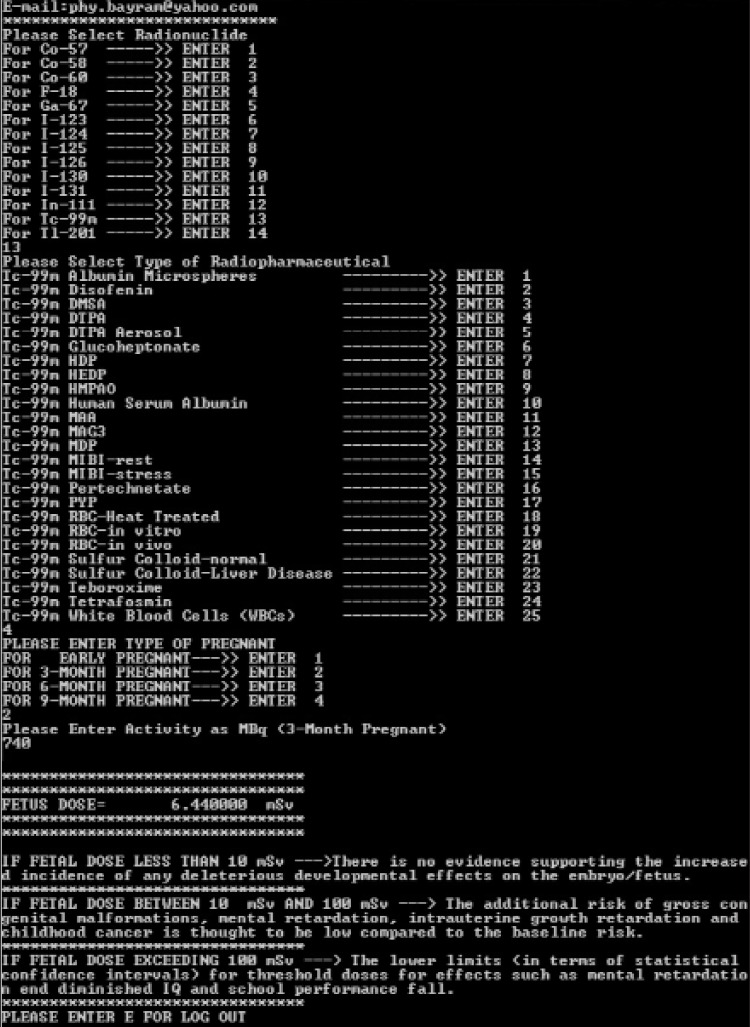
An example of calculation of approximate radiation dose to fetusfor renal function imaging with Tc-99m DTPA using nmfdose program

An example is presented in the figure (Figure 1) showing the calculation of approximate fetus dose for Tc-99m DTPA (diethylene triamine pentaacetic acid) which is a renal imaging agent and is widely used in clinical nuclear medicine. At the first step, Tc-99m radionuclide was selected via entering 13. At the second step, Tc-99m DTPA was selected via entering 4. At the third step, gestational age was selected as 3-month pregnant via entering 2 and finally activity of Tc-99m DTPA was selected as 740 MBq. The result of this calculation was obtained as 6.44 mSv. After the calculations, the program gives some suggestions relating to the level of received fetal dose.

Radioiodine (I-131) is another frequently used radionuclide for diagnostic purposes and for treating patients with hyperthyroidism and well differentiated thyroid cancer. Although this radionuclide is not allowed to be used for pregnant patients for treatment purposes, there might be some cases that physician and patient do not know about the pregnancy even after a pregnancy test. We therefore calculated the radiation dose to embryo for I-131 treatment using nmfdose, and we obtained it as 266.4 mSv. In this calculation, gestational age was selected as early pregnancy and the activity of the radionuclide was entered as 3700 MBq. The calculations of fetus doses mentioned before are summarized in Table 2. 

## DISCUSSION

Using nmfdose program, approximate embryo or fetus dose in pregnant patients for nuclear medicine applications can be easily calculated. On the other hand, there are some constraints about some special cases to be considered which are not included by nmfdose program for the pregnant patient. These cases are summarized as follows:

1. The fetus begins to take up iodine at around 10-13 weeks of gestation when the fetal thyroid is capable of concentrating iodine, which crosses the placenta and synthesize thyroid hormones (3,15). Because of this reason, calculation of the fetus dose does not depend on the value of % uptake for the stage of early pregnancy and nmfdose program which does not include the value of % uptake can be used for this stage. 

2. In thyroid cancer patients, a large amount of I-131 is often given to patients whose thyroid glands have been mostly removed surgically to destroy all the remaining thyroid tissue and the metastases. There may be a remnant of thyroid tissue, and/or some thyroid cancer metastases around the body. This situation should be taken into account when approximate fetal dose is calculated (16). As a result, except these cases, nmfdose program can be used. This program is useful and it provides a practical solution for the calculation of approximate fetus dose in nuclear medicine applications. It can be expanded easily for new dose estimations of other radiopharmaceuticals to fetus or more precise estimations than those of used in nmfdose are needed. nmfdose program can be obtained for free on request from the corresponding author.

## Figures and Tables

**Table 1:**
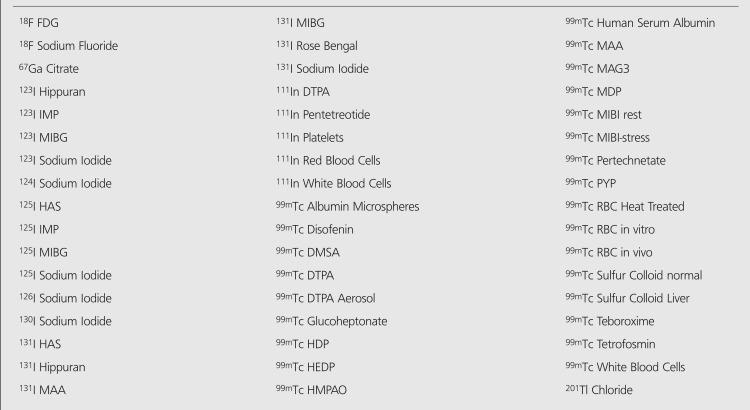
Some most commonly used radiopharmaceuticals included by nmfdose program

**Table 2:**
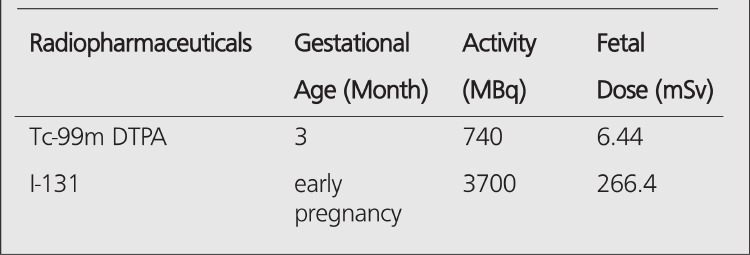
Results of fetus doses calculated by using nmfdose program

## References

[1] Streffer C, Shore R, Konermann G, Meadows A, Uma Devi P, Preston Withers J, Holm LE, Stather J, Mabuchi K (2003). H R. Biological effects after prenatal irradiation (embryo and fetus). Ann ICRP.

[2] Adelstein SJ (1999). Administered radionuclides in pregnancy. Teratology.

[3] Stather JW, Phipps AW, Harrison JD, Eckerman KF, Smith TJ, Fell TP, Nosske D (2002). Dose coefficients for the embryo foetus following intakes of radionuclides by the mother. J Radiol Prot.

[4] Steenvoorde P, Pauwels EK, Harding LK, Bourguignon M, Mariere B, Broerse JJ (1998). Diagnostic nuclear medicine and risk for the fetus. Eur J Nucl Med.

[5] (2000). International Commission on Radiological Protection. Pregnancy and medical radiation. Ann ICRP.

[6] Doll R, Wakeford R (1997). Risk of childhood cancer from fetal irradiation. Brit J Radiol.

[7] (1987). International Commission on Radiological Protection. Radiation dose to patients from radiopharmaceuticals..

[8] (1995). Age-dependent doses to members of the public from intake of radionuclides: Part 3. Ingestion dose coefficients. A report of a Task Group of Committee 2 of the International Commission on Radiological Protection.

[9] (1995). Age-dependent doses to members of the public from intake of radionuclides: Part 4. Inhalation dose coefficients. A report of a task group of Committee 2 of the International Commission on Radiological Protection.

[10] (1996). Age-dependent doses to members of the public from intake of radionuclides: Part 5. Compilation of ingestion and inhalation dose coefficients.

[11] (2001). Doses to the embryo and fetus from intakes of radionuclides by the mother. A report of The International Commission on Radiological Protection.

[12] Russell JR, Stabin MG, Sparks RB (1997). Placental transfer of radiopharmaceuticals and dosimetry in Pregnancy. Health Physics.

[13] Russell JR, Stabin MG, Sparks RB, Watson E (1997). Radiation absorbed dose to the embryo/fetus from radiopharmaceuticals. Health Phys.

[14] (2010). The Radiation Internal Dose Information Center official website available at:.

[15] Berkovski V, Eckerman KF, Phipps AW, Nosske D (2003). Dosimetry of radioiodine for embryo and fetus. Radiat Prot Dosimetry.

[16] Stabin MG, Watson EE, Marcus CS, Salk RD (1991). Radiation dosimetry for the adult female and fetus from iodine-131 administration in hyperthyroidism. J Nucl Med.

